# Orts- und zeitflexibles Arbeiten: Alte Geschlechterungleichheiten und neue Muster der Arbeitsteilung durch Digitalisierung

**DOI:** 10.1007/s41449-020-00213-y

**Published:** 2020-06-03

**Authors:** Tanja Carstensen

**Affiliations:** Institut für Soziologie, Ludwig-Maximilians-Universität München, Konradstraße 6, 80801 München, Deutschland

**Keywords:** Homeoffice, Geschlecht, Arbeit, Digitalisierung, Vereinbarkeit, Homeoffice, Gender, Work, Digital Transformation, Compatibility of Work and Family

## Abstract

Der vorliegende Beitrag verfolgt die Frage, inwiefern sich bei orts- und zeitflexiblem Arbeiten mit digitalen Technologien Vereinbarkeit von Beruf und Familie und Geschlechterungleichheiten in der häuslichen Arbeitsteilung verändern. Grundlage bilden Ergebnisse aus dem Forschungsprojekt „Wandel der Geschlechterverhältnisse durch Digitalisierung“ (Hans-Böckler-Stiftung). Es zeigen sich verschiedene Effekte: So ermöglichen mobiles Arbeiten und Homeoffice es Teilzeitbeschäftigten, ihre vertraglich vereinbarte Arbeitszeit zu erhöhen; flexible digitale Arbeit sorgt für emotionale und zeitliche Entlastungen bei spontanen Notfällen wie Krankheit der Kinder; aber auch der Umfang unsichtbarer und unbezahlter Mehrarbeit steigt. In der Regel kommt es dabei nicht zu einer Neuorganisation oder Umverteilung der unbezahlten Haus- und Sorgearbeiten. Dennoch zeigen sich in Ansätzen Verschiebungen der häuslichen Arbeitsteilung. Der Beitrag stellt die zentralen Ergebnisse des Projekts vor und diskutiert sie hinsichtlich ihrer Implikationen für Geschlechterungleichheiten.

*Praktische Relevanz*: Der Text gibt Aufschluss über aktuelle Erfahrungen mit orts- und zeitflexiblem Arbeiten aus Geschlechterperspektiven. Er liefert Hinweise auf förderliche und hinderliche Bedingungen für einen Abbau der Ungleichheiten in der Zuständigkeit für Beruf und Familie und der Arbeitsteilung in der Familie. Die Ergebnisse können für die Gestaltung betrieblicher Rahmenbedingungen und Regelungen orts- und zeitflexibler Arbeit genutzt werden.

## Abstract (en)

This paper follows the question to what extent the compatibility of work and family life and gender inequalities in the domestic division of labour change when working with digital technologies in a flexible manner in terms of time and place. It is based on the results of the research project “Changing gender relations through digital transformation” (Hans Böckler Foundation). Various effects have been observed: For example, mobile working and home office enable part-time workers to increase their contractual working hours; flexible digital work provides emotional and time relief in the event of spontaneous emergencies such as child illness; however, the extent of invisible and unpaid overtime is also increasing. In most cases, this does not lead to a reorganisation or redistribution of unpaid domestic and care work. Nevertheless, there are signs of shifts in the domestic division of labour. This article presents the central results of the project and discusses them with regard to their implications for gender inequality.

*Practical Relevance:* The text provides information on current experiences with flexible working times and places from a gender perspective. It provides indications of conducive and obstructive conditions for reducing inequalities in the responsibility for care work and in domestic division of labour. The results can be used for the design of conditions and regulations for flexible work in terms of time and place in companies.

## Einführung

Bereits seit den 1980er Jahren wird immer wieder die Erwartung formuliert, dass Technologien durch die Flexibilisierung von Arbeitsorten und Arbeitszeiten die Möglichkeiten für die Vereinbarkeit von Beruf und Familie erhöhen sowie zu einer Verringerung der Ungleichheit in der Arbeitsteilung von Frauen und Männern hinsichtlich Care- und Hausarbeit führen könnten. In der gegenwärtigen Arbeitswelt hat sich diese Flexibilisierung zugespitzt: Mit der zunehmenden beruflichen Nutzung von immer kleineren, digitalen Geräten und mobilem Internetzugang an fast allen Orten lässt sich mittlerweile die Normalisierung eines Arbeitens fast „immer und überall“ beobachten. Mit der Möglichkeit, die Erwerbsarbeit (noch weiter) aus den zeitlichen und räumlichen Strukturen zu lösen und flexibel an verschiedenen Orten und zu unterschiedlichen Zeiten zu arbeiten, könnten auch für Menschen mit Sorgeverpflichtungen – das heißt (nach wie vor) vor allem Frauen – neue Freiräume der Alltagsgestaltung entstehen. Zeitsouveränität, Reduzierung der Zeit für Arbeitswege und eine flexiblere Alltagsgestaltung ermöglichen, Erwerbsarbeitszeiten und -orte an die Bedürfnisse der Sorgearbeit anzupassen. Angemerkt sei dabei aus aktuellem Anlass, dass diese Möglichkeiten nicht mit der Situation zu verwechseln sind, die seit März 2020 angesichts der Corona-Pandemie einen großen Teil an Beschäftigten „auf einen Schlag“ ins Homeoffice versetzt hat und in der nach jahrelangen, eher zögerlichen Entwicklungen das Arbeiten von Zuhause nun sehr plötzlich zum Arbeitsalltag geworden ist. Diese sehr spezifische, neue Situation wird noch zu untersuchen sein, insbesondere welche Effekte der Umstand, dass sich durch die Schließung von Schulen und Kinderbetreuungseinrichtungen viele Eltern nun gemeinsam mit den zu betreuenden Kinder zuhause aufhalten und dabei die Anforderungen (gleichzeitiger) Erwerbsarbeit, Kinderbetreuung sowie „Homeschooling“ gestiegen sind, auf die Arbeitsteilung hat.

Der vorliegende Beitrag verfolgt die Frage, inwiefern sich bei orts- und zeitflexiblem Arbeiten mit digitalen Technologien Vereinbarkeit von Beruf und Familie und Geschlechterungleichheiten in der häuslichen Arbeitsteilung verändern. Die Ergebnisse beschränken sich aufgrund der Datenlage auf Personen, die in heterosexuellen Beziehungen leben, auch wenn dies bei Weitem nicht die einzige Familienkonstellation ist. Grundlage bilden Ergebnisse aus dem Forschungsprojekt „Wandel der Geschlechterverhältnisse durch Digitalisierung“ (Hans-Böckler-Stiftung). Es zeigen sich verschiedene Effekte: So ermöglichen mobiles Arbeiten und Homeoffice es Teilzeitbeschäftigten, ihre vertraglich vereinbarte Arbeitszeit zu erhöhen; flexible digitale Arbeit sorgt für emotionale und zeitliche Entlastungen bei spontanen Notfällen wie Krankheit der Kinder; der Umfang unsichtbarer und unbezahlter Mehrarbeit steigt. In der Regel kommt es dabei allerdings nicht zu einer Neuorganisation oder Umverteilung der unbezahlten Haus- und Sorgearbeiten. Dennoch zeigen sich in Ansätzen Verschiebungen der häuslichen Arbeitsteilung. Der Beitrag stellt die zentralen Ergebnisse des Projekts vor und diskutiert sie hinsichtlich ihrer Implikationen für Geschlechterungleichheiten. Zunächst werden hierfür einige Überlegungen sowie der Forschungsstand zu Gender, Arbeit, Digitalisierung und Homeoffice skizziert. Nach einem kurzen Überblick über das methodische Vorgehen werden verschiedene Fälle exemplarisch vorgestellt und diskutiert.

## Ungleiche Arbeitsteilungen und die Digitalisierung als Anlass für Neuverhandlungen

### Fortbestehende Geschlechterungleichheiten in der Arbeitswelt

Arbeit ist bis heute hochgradig vergeschlechtlicht. Trotz gestiegener Frauenerwerbstätigkeit und Gleichheitsentwicklungen ist Erwerbsarbeit nach wie vor vertikal und horizontal nach Geschlecht segregiert (Aulenbacher 2010). Zum einen finden sich mehr Männer als Frauen in hochbezahlten Arbeitsverhältnissen und Führungspositionen (Holst und Kirsch 2016). Zum anderen ist der Arbeitsmarkt segregiert nach sogenannten Männer- und Frauenberufen, mit ungleichen Bewertungen hinsichtlich gesellschaftlicher Anerkennung und Bezahlung.

Vor allem aber ist die Arbeitsteilung zwischen den Geschlechtern hinsichtlich bezahlter und unbezahlter Arbeit ungleich. Die Erwerbsquote lag 2016 bei Frauen bei 73,4 %, bei Männern bei 81,7 % (Bundesagentur für Arbeit 2018, S. 5). Zwar hat sich der Abstand hier in den letzten Jahrzehnten deutlich verringert, allerdings bestehen hinsichtlich des Umfangs der wöchentlichen Arbeitszeit nach wie vor deutliche Unterschiede: Teilzeit arbeiteten in 2017 7,1 Mio. Frauen, aber nur 1,9 Mio. Männer. Bemerkenswert ist zudem, dass die Anzahl der Vollzeit erwerbstätigen Frauen seit 2007 nicht gestiegen ist, sondern nach wie vor bei 7,8 Mio. liegt (Bundesagentur für Arbeit 2018, S. 9). So ist es wenig verwunderlich, dass unter Eltern das Familienmodell aus Vollzeit arbeitenden Vätern und Teilzeit arbeitenden Müttern in der Bundesrepublik das am weitesten verbreitetste ist (2012: 38,4 %; statista 2014), gefolgt von 29,4 %, bei denen nur der Vater erwerbstätig ist und erst dann mit 13,8 % Paare, in denen beide Vollzeit erwerbstätig sind. Dass Frauen in Partnerschaften mit Kindern mehr arbeiten als Männer, kommt nur 6,3 % vor, davon arbeiten in 5,3 % der Fälle nur die Frauen, in 1 % der Fälle arbeiten die Mütter Vollzeit und die Väter Teilzeit. Und auch nur bei 1,6 % arbeiten beide Teilzeit (statista 2014). Neben diesen Ergebnissen zur Aufteilung der Arbeit in heterosexuellen Partnerschaften ist die Datenlage zur Arbeitsteilung in nicht-heterosexuellen Beziehungskonstellationen insgesamt schwach (Wimbauer und Motakef 2019, S. 1107), wobei einiges darauf hinweist, dass auch hier die unbezahlte Arbeit nicht gleich verteilt (u. a. Funcke und Thorn 2010, S. 24 f), in vielen Fällen aber egalitärer als in heterosexuellen Partnerschaften ist (Buschner 2014, S. 247).

Insgesamt sind deutlich mehr Frauen mit den unbezahlten Arbeiten, d. h. vor allem Kinderbetreuung und Hausarbeit, zunehmend aber auch Pflege von Angehörigen, beschäftigt (Statistisches Bundesamt 2015; Winker 2015). Selbst in Partnerschaften mit Kindern, in denen beide Vollzeit arbeiten, verwenden Frauen deutlich mehr Zeit auf Kinderbetreuung und Haushalt als Männer (Hobler et al. 2017). Vereinbarkeit von Beruf und Familie ist damit noch immer vor allem die Aufgabe von Frauen. Insgesamt verschärfen sich zudem seit Jahren nicht nur die Anforderungen und Belastungen im Bereich der Erwerbsarbeit, sondern auch die Bedingungen für gute Sorgearbeit. Gründe hierfür sind zum einen steigende Ansprüche an Kindererziehung, zum anderen erschwerte Rahmenbedingungen durch Privatisierungen und staatliche Einsparungen, z. B. für Pflege und im Gesundheitsbereich, die individuell bzw. im Privaten aufgefangen werden müssen (vgl. Winker und Carstensen 2007; Winker 2015).

Gleichzeitig sind die Entwicklungen höchst widersprüchlich (u. a. Lenz et al. 2017). So finden sich neben diesen resistenten Ungleichheiten diverse Gleichheitstendenzen. In Unternehmen steigt die Nachfrage nach Kompetenzen, die als „weiblich“ gelten, Geschlechterrollen werden flexibilisiert, Männer als neue Väter adressiert, und auch betriebliche Ansätze zu Gleichstellung und Diversity verweisen auf eine Öffnung und Sensibilisierung für Gender-Themen – und zeigen gleichzeitig auch die ökonomische Verwertbarkeit dieser Vielfalt.

### Technik und die Möglichkeit für Neuverhandlungen

Auch Technik war und ist bis heute tief vergeschlechtlicht und dabei in vielerlei Hinsicht eine Männerdomäne. Stereotype Zuschreibungen von Kompetenzen und Fähigkeiten und hartnäckige Vorstellungen wie eine vermeintliche Technikdistanz von Frauen prägen den Zugang zu und die Nutzung von Technik maßgeblich (Cockburn 1988; Wajcman 1994). Historisch hat sich aber auch immer wieder gezeigt, dass es mit der Einführung neuer Technologien zu Umbewertungsprozessen von Arbeit kommen kann. Wajcman (2004) weist darauf hin, dass jede neue Technologie immer auch Anlass sein kann, Geschlechterverhältnisse neu zu verhandeln, Instabilitäten in Machtverhältnisse zu bringen und beispielsweise Rollenzuschreibungen und Arbeitsteilungen in Bewegung zu setzen (Carstensen 2015, 2019; Kutzner 2018).

Dass auch die Frage nach Vereinbarkeit von Beruf und Familie und häuslicher Arbeitsteilung dabei keine neue ist, sondern in Phasen technologischen Wandels immer wieder gestellt wurde und wird, zeigt zum Beispiel ein Rückblick auf die frühen Debatten um das Internet in den 1990er Jahren. So stellte sich hinsichtlich des Internets bereits die Frage, inwiefern mit den damit verbundenen Möglichkeiten auf flexible Gestaltung von Arbeitszeit und Arbeitsort auch neue Chancen für verschiedene Lebensentwürfe von Frauen, neue Geschlechterarrangements, Arbeitsteilungen und höhere Lebensqualität verbunden sind (u. a. Winker 1997). Mit einer erwarteten Erosion des Normalarbeitsverhältnisses ließen sich Hoffnungen auf einen Abbau geschlechtshierarchischer Arbeitsteilungen verbinden.

### Homeoffice – Ambivalente Effekte auf Geschlechterungleichheiten

Mittlerweile liegen zur Frage, wie sich Vereinbarkeit von Beruf und Familie und häusliche Arbeitsteilung verändern, wenn ein Teil der Erwerbsarbeit im Homeoffice geleistet wird, verschiedenste Forschungsergebnisse vor. Zunächst ist – trotz aller technologischen Möglichkeiten – festzuhalten, dass gerade in Deutschland nach wie vor viele Erwerbstätige nicht frei in der Wahl ihres Arbeitsorts sind. Zumindest unter den abhängig Beschäftigten sind es hierzulande nur 12 %, die überwiegend oder gelegentlich von zu Hause aus arbeiten, obwohl dies bei 40 % der Arbeitsplätze möglich wäre und insgesamt ungefähr jede fünfte Person die Gelegenheit ergreifen würde, wenn sie das Angebot erhalten würde (Brenke 2016). Es sind eher Beschäftigte in höheren beruflichen Stellungen, die Zugang zum Homeoffice haben (Lott 2016). Oftmals werden gerade Frauen zudem von unternehmenskulturellen Barrieren an den Möglichkeiten ortsflexiblen Arbeitens gehindert (Lott und Abendroth 2019).

Insgesamt sind die Forschungsergebnisse widersprüchlich (Pfeiffer 2012). Laut dem DGB-Index Gute Arbeit (2016, S. 11) stellen 68 % der Beschäftigten keinen Effekt auf die Work-Life-Balance durch Digitalisierung fest. Wajcman et al. (2010) kommen zu dem Ergebnis, dass eher private Nutzungsweisen in die Erwerbsarbeit hinein reichen als umgekehrt und dass das kurze Erledigen von außerberuflichen Aufgaben zu einer besseren Vereinbarkeit der Anforderungen in beiden Bereichen führen kann.

Positive Effekte auf die Vereinbarkeit haben beispielsweise das Einsparen von Wegezeiten sowie die Möglichkeit, berufliche Anforderungen und Anforderungen aus Care-Verpflichtungen besser aufeinander abstimmen zu können. Arntz et al. (2019) zeigen, dass Mütter in Teilzeit durch diese Zeitgewinne ihre vertraglich vereinbarte Arbeitszeit durch Homeoffice erhöhen können. Damit verringert sich der gender gap hinsichtlich Arbeitszeit und Monatseinkommen durch flexibles Arbeiten. Gleichzeitig kommt die Studie allerdings auch zu dem Befund, dass Homeoffice bei Vätern dazu führt, dass ihr Stundenlohn ansteigt, bei Müttern hingegen nicht.

Hinsichtlich der häuslichen Arbeitsteilung zwischen Männern und Frauen durch zeit- und ortsflexibles Arbeiten zeigen sich eher Verfestigungen ungleicher Muster: Lott (2019) kommt beispielsweise auf Grundlage der Daten des sozio-ökonomischen Panels zu dem Ergebnis, dass Mütter und Väter flexible Angebote wie Homeoffice unterschiedlich nutzen. Väter nutzen Homeoffice und selbstbestimmte Arbeitszeiten hiernach, um deutlich länger zu arbeiten; sie machen mehr Überstunden als Väter, die nur in der Firma arbeiten. Mütter mit den gleichen Möglichkeiten arbeiten zwar ebenfalls länger, jedoch nicht im gleichen Maße. Vielmehr nutzen sie Homeoffice deutlich mehr für Kinderbetreuung, sie investieren auch mehr Zeit in Kinderbetreuung als Mütter, die nie im Homeoffice arbeiten (auch Lott und Chung 2016). Zum gleichen Ergebnis kommen auch Schmook und Kondradt (2000), und auch viele internationale Studien verweisen darauf, dass Frauen bei Homeoffice mehr Zeit für Hausarbeit und Kinderbetreuung aufwenden und sich damit ungleiche Muster der Arbeitsteilung verfestigen (u. a. Sullivan und Lewis 2001; Hilbrecht et al. 2013; Powell und Craig 2015; Kim 2018). Maus und Winker (2001) wiederum zeigen, dass Telearbeit durchaus zu leichten Verschiebungen der Verantwortlichkeiten in Richtung Gleichheit zwischen den Partner*innen führen kann.

Interessant in diesem Zusammenhang sind auch die ersten geschlechtersoziologischen Einordnungsversuche der Effekte des Corona-bedingten Arbeitens im Homeoffice, insbesondere angesichts der besonderen Situation, Homeoffice mit Kinderbetreuung und „Homeschooling“ zu vereinbaren: Einiges spricht dafür, dass die Corona-Krise Geschlechterungleichheiten zuspitzt (Carstensen et al. 2020). Lewis (2020) geht beispielsweise davon aus, dass Frauen die Hauptlast der nach Hause verlagerten Care-Arbeit übernehmen: „[E]ine besonders frappierende Folge des Coronavirus ist, dass er viele Paare in die fünfziger Jahre zurückkatapultiert“. Genauso gut könnte es aber auch sein, dass die Selbstisolation Paare dazu zwingt, sich gegenseitig in den alltäglichen Routinen zu beobachten und wahrzunehmen. Dabei würden die für die Partner*in häufig unsichtbare Haushaltsarbeit, Kinderbetreuung und die damit verbundenen Belastungen sichtbar (McNaughton 2020).

### Neuverhandlungen durch Digitalisierung?

In Bezug auf die Digitalisierung werden erneut Hoffnungen auf eine bessere Vereinbarkeit von Beruf und Familie formuliert. Dies spiegelt sich nicht zuletzt in den medialen, politischen und unternehmerischen Diskursen wider, die die Verbreitung digitaler Technologien seit einigen Jahren intensiv begleiten. Das Bundesministerium für Bildung und Forschung (2015: § 23) betont beispielsweise, dass die „technischen Möglichkeiten der digitalen Arbeit […] neue Chancen der zeitlichen, räumlichen und organisatorischen Flexibilität“ eröffnen: „Arbeitsformen wie Vertrauensarbeit, Home Office, mobiles und flexibles Arbeiten gewinnen zunehmend an Bedeutung. Social Collaboration, Open Innovation, virtuelle Präsenz lassen die Grenzen zwischen Arbeit und Freizeit fließend werden. Die digitale Vernetzung kann dadurch den Wünschen vieler Beschäftigter entgegenkommen, denn sie bietet die Chance zur Erhöhung des selbstbestimmten Handelns sowie der besseren Vereinbarkeit von Arbeit und Freizeit, Familie und Beruf“ (Bundesministerium für Bildung und Forschung 2015, § 23).

Mit der Möglichkeit, die Erwerbsarbeit (noch weiter) aus den zeitlichen und räumlichen Strukturen zu lösen und flexibel jederzeit und von überall zu arbeiten, könnten auch für Menschen mit Sorgeverpflichtungen – das heißt vor allem Frauen – somit weitere Freiräume der Alltagsgestaltung entstehen (Wischermann und Kirschenbauer 2015). Zeitsouveränität, Reduzierung der Zeit für Arbeitswege und eine flexiblere Alltagsgestaltung ermöglichen, Erwerbsarbeitszeiten und -orte an die Bedürfnisse der Sorgearbeit anzupassen. Arbeiten können mit nach Hause oder möglicherweise sogar mit auf den Spielplatz genommen werden; Büroarbeitszeiten können um weitere Arbeitszeiten ergänzt werden.

Dass orts- und zeitflexibles Arbeiten erneut an Relevanz gewinnt, hat allerdings nicht nur technologische Ursachen – wenngleich sich zeigt, dass im Vergleich zu früheren Erfahrungen mit digitaler und mobiler Arbeit oftmals erst jetzt die Verbindung über Internet, mobile Geräte und userfreundliche Software ins Büro stabil und verlässlich genug geworden zu sein scheint. Ton- und Bildqualitäten von Konferenz- und Chatsoftware, starkes W‑LAN und digitale Kommunikationswege, die auch Datensicherheit gewährleisten, werden als absolut notwendige Voraussetzungen für das reibungslose Funktionieren mobiler und digitaler Arbeit beschrieben, die in vielen Branchen erst allmählich Standard werden. Aber auch Kosteneinsparungen durch Büroraumreduzierungen, die oftmals vermischt werden mit (Mode‑)Themen wie „agiles Arbeiten“ haben zur Bedeutungssteigerung von Homeoffice und mobiler Arbeit beigetragen. Zudem sind die Ansprüche der Beschäftigten an flexibleres Arbeiten gestiegen; diese zu befriedigen, ist zudem Teil von Strategien zur Erhöhung der Arbeitgeberattraktivität sowie der Motivation der Beschäftigten geworden. Mobile Arbeit und Homeoffice haben hierbei an Dringlichkeit gewonnen, zum einen aufgrund der Wünsche der Beschäftigten nach einer souveräneren Alltagsgestaltung und besserer Vereinbarkeit von Beruf und Familie – und hierbei geht es nicht nur um Kinderbetreuung, sondern zunehmend auch um die Pflege von Angehörigen. Zum anderen wird auch der Zeitaufwand durch lange Pendelwege und -zeiten immer öfter als Belastung wahrgenommen.

## Methodik

Das Forschungsprojekt „Wandel der Geschlechterverhältnisse durch Digitalisierung“ (gefördert von der Hans-Böckler-Stiftung, 2018–2020) verfolgt die Fragestellung, inwiefern sich mit der Nutzung von digitalen Technologien und den damit verbundenen Änderungen und Neuregelungen der Arbeitsorganisation in der konkreten betrieblichen Praxis sowie im Alltag neue genderrelevante Veränderungen erkennen lassen. Hierfür wurden Betriebsfallstudien und Einzelinterviews mit Beschäftigten, Betriebsräten, Gleichstellungs- und Diversitybeauftragten sowie Personalverantwortlichen geführt. Die untersuchten Unternehmen und befragten Personen zeichnen sich dadurch aus, dass sie digitale und mobile Technologien (insbesondere Smartphone, Laptop, Internet, Social Media sowie Collaboration-Software) nutzen und zumindest teilweise die Möglichkeit zu zeitlich und räumlich flexibler Arbeit haben. Dabei wurden insbesondere Eltern interviewt. Insgesamt wurden 49 qualitative Leitfaden-Interviews mit 50 Personen geführt. Die Betriebsfallstudien wurden in folgenden Branchen durchgeführt: Automobilbranche (12 Interviews), Transport (18 Interviews), IT-Startup (3 Interviews), Forschung (3 Interviews), Weiterbildung (3 Interviews). Einzel-Interviews wurden geführt mit Personen aus Medizin, Recht, Beratung, Banken, Versicherungen und Bäckerei. Interviewt wurden 31 Frauen (davon 6 Betriebrätinnen und 4 Frauen, die im Bereich Gleichstellung/Diversity arbeiten) sowie 19 Männer (davon 5 Betriebsräte und ein Mann, der im Bereich Diversity arbeitet). Die Interviews wurden mit der qualitativen Inhaltsanalyse (Mayring 1997) ausgewertet.

Für den vorliegenden Text wurden exemplarisch Passagen und Fälle aus Interviews mit Beschäftigten in heterosexuellen Paarbeziehungen ausgewählt, die Aufschluss darüber geben, inwiefern und unter welchen Bedingungen sich mit digitalem orts- und zeitflexiblen Arbeiten Vereinbarkeit von Beruf und Familie und Geschlechterungleichheiten in der häuslichen Arbeitsteilung verändern. Diese werden nun im Folgenden vorgestellt.

## Ergebnisse

Die Interviews zeigen, dass im Kontext der Digitalisierung Arbeit und Arbeitsteilung zum Teil neu gestaltet werden. Dies betrifft auch die Geschlechterverhältnisse. Die Auswirkungen sind dabei unterschiedlich, wie im Folgenden erörtert wird.

### Erhöhung der Arbeitszeit von Frauen in Teilzeit

Einige der interviewten Frauen betonen zunächst, dass sie ihr Alltagsarrangement aus Erwerbsarbeit und Zeit für Kinderbetreuung nicht überstrapazieren möchten. Auch wenn sie gern erwerbstätig sind, kommt zum Ausdruck, dass ihnen Zeit mit den Kindern ebenfalls sehr wichtig ist. Das ist der Grund dafür, dass sie Teilzeit arbeiten. So beschreibt eine Angestellte mit zwei Kindern:

also 28 h für mich mit zwei Kindern ist – mehr würde ich jetzt glaube ich nicht, im Moment erst mal nicht machen. […] Es wird sich wahrscheinlich wieder regeln, wenn der Sohn dann auch in die Kita geht […] wobei mir der Freitagstag eigentlich auch heilig ist und es ganz schön ist, mal einen Tag nur was mit den Kindern zu machen und auch mal wieder – genau, nicht von der Arbeit zu stressen, nach Hause zu kommen und zu gucken, ob man noch was macht.

Zusammen mit Wegezeiten, Verkehrsproblemen und der Erfahrung, dass durch Krankheit der Kinder oftmals der Alltag durcheinandergerät, beschreiben einige der Interviewten ihre zeitlichen Möglichkeiten für Erwerbsarbeit als begrenzt, wie beispielsweise diese Angestellte, die ein Kind hat und deren Arbeitsweg durch ein verkehrstechnisch belastetes Nadelöhr führt:

Genau, das ist für mich ein ganz großer Punkt, weil ich die [Name eines Verkehrsabschnitts] kreuzen muss. Das heißt, das war einer der Gründe, warum ich immer gesagt habe, freitags würde ich lieber nicht arbeiten.

Auch in meinen Daten zeigt sich dann, wie Arntz et al. (2019) bereits quantitativ belegt haben, dass die Möglichkeit zu mobiler Arbeit und Homeoffice es Teilzeitbeschäftigten erlaubt, ihre vertraglich vereinbarte Stundenzahl in solchen, eng strukturierten Alltagsarrangements, zu erhöhen. Das ermöglicht oftmals, nicht nur mehr zu arbeiten, sondern auch eine interessantere Tätigkeit mit mehr Entwicklungsmöglichkeiten ausüben zu können. Eine Interviewte ist von Teilzeit auf Vollzeit – und damit auf eine interessantere Tätigkeit – gewechselt, als ihr mobile Arbeit angeboten wurde. Dies wurde für sie mit ihrem Kind und einem langen Arbeitsweg nur möglich – ohne, „dass ich mich völlig zerreißen muss“, weil sie nun teilweise zuhause arbeitet.

Mir wurde das angeboten im Rahmen eines Jobwechsels. […] Also man hat mir gesagt, wenn du – wenn man gerne umsteigen – wenn der Bedarf wäre bei mir, wieder Vollzeit zu kommen und ich mich – mir den Job vorstellen könnte, dann könnte man das durchaus arrangieren, dass man in dem Rahmen auch eine Stelle etablieren kann, die mir entgegenkommt, weil ich halt diese Fahrprobleme auch angebracht habe, die genau damals auch aktiv waren. Also ich habe durchaus über eine Stunde nach Hause gebraucht.

Frauen mit Kindern, die möglichweise defensiv eine geringere Stundenzahl vertraglich vereinbaren würden, um ihr Alltagsmanagement nicht überzustrapazieren, werden dabei teilweise gerade von Betriebsrat oder Gleichstellungsbeauftragten bewusst motiviert, etwas mehr zu arbeiten und dadurch Aufstiegschancen zu erhöhen, interessantere Tätigkeiten auszuüben und ihre Rente zu verbessern. Eine Betriebsrätin sagt: „So, und ganz ehrlich, wenn ich Frauen berate während der Schwangerschaft, so nach dem Motto, welche Karriereoptionen willst du? Wie stellst du dir den Wiedereinstieg vor, ist meine Empfehlung immer, so viel Stunden wie möglich.“

An dieser ersten Konstellation zeigt sich, dass orts- und zeitflexibles Arbeiten die Ungleichheiten zwischen den Geschlechtern hinsichtlich des Umfangs bezahlter Erwerbsarbeit durchaus verringern kann. Dies hat möglicherweise Auswirkungen auf Karrieremöglichkeiten, in jedem Fall aber auf materielle Absicherung und die Verringerung von geschlechtertypischen Risiken wie Altersarmut.

### Entspannung, zeitliche und emotionale Entlastung

Ein weiterer wichtiger Effekt der digital gestützten Möglichkeiten flexibler Arbeit ist, dass einige der interviewten Frauen mit Kindern betonen, dass allein das Wissen um die Möglichkeit, im Notfall zuhause bleiben zu können, ohne dass es ein größeres Problem ist, ihren Alltag deutlich entspannt, und das sowohl zeitlich als auch emotional. Angebote, flexibel arbeiten zu dürfen, mindern den Stress und Druck beispielsweise in Situationen, wenn die Kinder krank werden oder andere ungeplante Ereignisse eintreten. An dem folgenden Zitat wird deutlich, wie belastend es sein kann, spontane Lösungen für Situationen zu finden, in denen die Vereinbarkeit von Beruf und Familie zusammenbricht, wie es eine weitere Angestellte mit zwei Kindern beschreibt:

Man hat eigentlich – ja, also man hat mehr Ruhe, ne? Also man hat ein besseres Gefühl, wenn man in dem Moment merkt, oh Scheiße, das Kind ist krank. Dann weiß ich im Hinterkopf, ach ich = ich muss jetzt nicht gucken, wie ich meine Mutter organisiert kriege oder irgendeinen Babysitter. Ich weiß sofort, okay, ich habe die Möglichkeit. So, und dann ist man eigentlich auch – geht man halt viel gelassener mit der Situation dann direkt um, ne?

Bemerkenswert ist in diesem Zitat und vielen ähnlichen Schilderungen in anderen Interviews mit Frauen aber auch, dass die Väter jeweils kaum oder gar nicht vorkommen. Die Verantwortung und Aufgabe, die Situation mit dem kranken Kind zu lösen, wird selbstverständlich akzeptiert; Rollenzuschreibungen werden an dieser Stelle nicht in Frage gestellt. Gleichzeitig scheint auch die Inanspruchnahme der gesetzlichen Möglichkeit, sich wegen Krankheit des Kindes freistellen zu lassen, dem Anspruch und der Anforderung, immer zu arbeiten und ansprechbar zu sein, zu weichen.

Wichtig ist hierbei eine Differenzierung der unterstützenden Rahmenbedingungen: Die digitalen Technologien ermöglichen Flexibilität und müssen, wie schon erwähnt, stabil funktionieren. Viel entscheidender für die emotionale Entlastung scheint aber gegenseitiges Verständnis für solche Situationen in den einzelnen Abteilungen zu sein sowie eine gute Kommunikation mit den Vorgesetzten. Ist dies gegeben, wird das Homeoffice mit krankem Kind als unkompliziert und entlastend beschrieben, wie die bereits zitierte Interviewte beschreibt:

Ich habe zwei kleine Kinder, die sind immer irgendwie krank. […] Ja, typische Situation, wir wachen morgens auf und mein Großer hat Fieber. Ja, dann schreibe ich eine Whatsapp. [lacht] […] an meinen Vorgesetzten […] sage hier, [Name Sohn] hat Fieber, ich würde gerne Homeoffice machen. Und er sagt, ja, geht klar. Dann wähle ich mich von zu Hause ein, da gibt es so ein – so ein, ich weiß gar nicht, wie es heißt, Connector irgendwas, da gibt man ein Passwort ein und bekommt dann so eine Nummer, dann wählt man sich da ein und dann ist man eigentlich im [Firmen]-Netz. So, und dann ist, das fiebernde Kind liegt dann auf der Couch, und ich sitze dann daneben mit meinem Laptop und arbeite.

Ist gegenseitiges Verständnis für Vereinbarkeitsherausforderungen gegeben, sind auch Störungen und Situationen, in denen klare Grenzziehungen nicht gelingen, kein Problem, wie eine Interviewte schildert:

Mein Chef hat auch Kinder, auch ein kleines Kind. Meine Kollegin hat drei Kinder. Das Verständnis ist da, ne, dass halt vielleicht auch mal ein Kind dann reinplappert, wenn man gerade in einer Audio ist oder so, es geht halt nicht anders. Ist dann einfach so. Aber das ist in Ordnung. Zumindest bei uns, ne?

Deutlich wird an dieser Konstellation, dass keine Hinweise auf grundlegende Veränderungen in der Arbeitsteilung von Frauen und Männern zu finden sind, dass aber einzelne Belastungs- und Stressfaktoren in der Vereinbarkeit von Beruf und Familie (für Mütter) reduziert werden können. Die Technik ist dabei eine Grundlage, die Verbindung zum Büro herzustellen; klare Regelungen, verständnisvolle Vorgesetzte und eine familienfreundliche Unternehmenskultur sind aber vermutlich entscheidender.

### Pragmatische Lösungen: Zuhause bleibt, wer den flexibleren Job hat

Während bis hierhin vor allem betrachtet wurde, was sich verändert, wenn Frauen orts- und zeitflexibel arbeiten, wird nun im Folgenden eine Konstellation beschrieben, in der ein männlicher Beschäftigter mit zwei Kindern die Möglichkeit hat, im Homeoffice zu arbeiten, während dies seiner Frau nicht möglich ist. Der Interviewte argumentiert hier pragmatisch, dass er auch wegen der Kinder häufig zuhause arbeitet, weil es „praktisch“ ist.

Ja oft, ich habe zwar – meine Frau ist berufstätig und habe zwei Kinder und die sind schulpflichtig und da ist halt manchmal so von der Orga her ist es einfach praktischer, da flexibel drauf reagieren zu können. Keine Ahnung, der Kindergarten ist zu, die machen einen Betriebsausflug und dann ist man halt dann zu Hause an dem Tag und betreut die Kinder.

Wichtig ist auch hier die verständnisvolle Unternehmenskultur, die es dann auch ermöglicht, Situationen zu überbrücken, in denen er für die Erwerbsarbeit und gleichzeitig für die Kinder ansprechbar sein muss:

Aber da haben auch die Kollegen teilweise Verständnis. Also wir machen es schon bestimmt zwei Jahre so, und ein – mein Sohnemann, der ist relativ klein, der hat immer dann früher mal im Hintergrund gewinselt, geweint und – ja, das funktioniert. Die meisten, die dann anrufen, denen kann man das kurz erklären, dass man im Homeoffice ist, die haben da volles Verständnis für.

### Unsichtbare Mehrarbeit und Vereinbarkeitsleistungen

Dass orts- und zeitflexibles Arbeiten dazu führt, dass Überstunden geleistet werden, ist bekannt. Deutlich wird auch in den Interviews an vielen Stellen, dass im Homeoffice insgesamt mehr gearbeitet wird. Eine Interviewte begründet dies mit der eingesparten Wegezeit:

Der Aufwand ist es, ne? Man spart – also ich spare eine Stunde, also morgens halbe und abends halbe Stunde Fahrzeit. Die arbeitet man länger. Also ich kann definitiv sagen, dass ich zu Hause mehr arbeite als hier. Und ja, das heißt, man – dieser ganze Stress, ich meine das immer in Anführungszeichen jetzt bitte, es ist ja jetzt – klingt jetzt ein bisschen [lachend:] albern vielleicht. Aber mit = mit Anziehen, mit = mit fertig machen, man sitzt halt einfach zu Hause und = und = und es ist einfach

Auch andere beschreiben, dass sie zuhause „durchaus ein bisschen mehr“ schaffen. Oftmals wird der Homeoffice-Tag sogar dazu genutzt, besonders viel abzuarbeiten: „Da bin ich eigentlich auch froh darum, weil man bekommt halt mehr erledigt an dem Tag dann. […] Also man weiß, das ist der Tag, wo man viel Mengen, also Quantität weggearbeitet bekommt.“

Daneben ist noch ein weiterer Aspekt relevant, der zeigt, inwiefern digitale und mobile Technologien dazu führen, dass immer mehr Situationen als Erwerbsarbeitszeit nutzbar gemacht werden, ohne dass dies – innerbetrieblich oder gesellschaftspolitisch – sichtbar wird, und damit auch darauf verweist, welches Ausmaß Arbeitsverdichtung und die kaum in der Arbeitszeit zu bewältigende Arbeitsmenge angenommen haben. Eine Studie aus Island, die Führungskräfte interviewt hat, zeigt, dass auch die Zeit mit Kindern, u. a. am Wochenende, nach Möglichkeit für Erwerbsarbeit genutzt wird. Dort beschreibt ein Manager mit zwei Kindern die Situation am Wochenende folgendermaßen: „‚It is important for me to work on the sofa, but not in a separate room. That is actually the only way for me to use the three hours I have with my kids also for work‘“ (Rafnsdottir und Juliusdottir 2018, S. 87).

Deutlich wird hier die Tendenz, beinahe jede Situation als Erwerbsarbeitszeit verfügbar zu machen, dadurch mehr Arbeit erledigen und so den Alltag optimieren zu können. Dies kann, wie bereits beschrieben, zu einer Normalisierung der gleichzeitigen Erfüllung von Erwerbsarbeits- und Sorgearbeiten führen. Digitale Technologien erweisen sich dabei als Hilfsmittel, die gestiegenen Anforderungen in allen Bereichen besser zu bewältigen und über Multitasking, permanente Erreichbarkeit und das ständige Erledigen von Erwerbsarbeitsaufgaben zwischendurch mehr schaffen zu können. Probleme der Vereinbarkeit bzw. die Unvereinbarkeit von Erwerbsarbeitsansprüchen mit anderen Lebensbereichen werden damit noch stärker als bisher individualisiert gelöst – weil es technisch möglich ist – und damit als gesellschaftlich und betrieblich zu lösende Probleme dethematisiert (auch Carstensen 2019). Wie ambivalent sich dies für die Einzelnen teilweise darstellt, verdeutlicht das folgende Zitat einer Angestellten:

Dann kann ich mich abends in Ruhe noch mal dran setzen. […] Und dann klappt das ganz gut. Bleibt dann meistens auch nicht bei kurz, aber – [lacht] […] wenn man dann dran sitzt, ist das ja doch meistens so, dass es länger wird, aber nein, das = das klappt ganz gut. […]. Also es ist schon anstrengend, ne? Ich = ich merke auch, dass – am Freitag ist immer so der Tag, wo ich dann zu Hause bin, wo man dann echt kaputt ist. Aber es ist eigentlich ganz gut. Also ich finde die Balance auch eigentlich = eigentlich recht gut. Alleine dadurch, dass – also ich verbringe super gerne Zeit mit meinen Kindern, keine Frage, aber ich finde es halt auch schön, einfach mal zwischendurch was anderes zu machen.

Digitales orts- und zeitflexibles Arbeiten befördert die weitere Ausweitung von Erwerbsarbeit in viele Bereiche des Alltags – womit einerseits gestiegene Anforderungen abgefedert werden können, andererseits auf Dauer neue Belastungen entstehen. Dies geschieht in vielen Situation unbemerkt, unsichtbar und unbezahlt.

### Neue Anforderungen an die Herstellung von Sichtbarkeit und Kontrollierbarkeit

Als weitere Anforderung zeigt sich in den Interviews, dass bei Nicht-Anwesenheit im Büro das Gefühl besteht, bewusst zu signalisieren, dass man arbeitet:

Ich melde mich auch relativ häufig bei meinen Kollegen, weil ich Fragen zu [Produkten] habe, zu bestimmten Fallbeispielen. Und die sehen und merken, ich bin permanent in diesen Themen drin. Das bedeutet, die wissen, wenn ich da jetzt anrufe oder sehen zum Beispiel auch über Link, sie ist grün, sie ist online, dann = dann weiß sie, was ich möchte. Ich schicke denen E‑Mails zu, in denen ich Rückfragen beantwortet haben möchte oder vielleicht Dinge weiter delegiere. Also sie kriegen schon mit, dass ich an Bord bin und auch aktiv.

Als neue Anforderung mobilen, digitalen Arbeitens kommt offensichtlich hinzu, die eigenen Aktivitäten und Arbeitsleistungen offensiv zu zeigen, und damit Sichtbarkeit und Kontrollierbarkeit herzustellen. Dies wird unterstützt durch eigene Ansprüche an die Arbeit und den Wunsch, diese „gut“ zu machen, so dass einige es als Herausforderung beschreiben, die digitalen Geräte nicht dafür zu nutzen, außerhalb der Arbeitszeit Arbeitsangelegenheiten zu bearbeiten. Arbeit wird als „Versuchung“ empfunden: „Und dann nicht zu sagen, ich mache das jetzt mal eben noch, empfinde ich durchaus als [lachend:] eine Versuchung. […] Weil ich einfach auch daran interessiert bin, meinen Job gut zu machen. […] Ich finde meinen Job spannend, ich mag den gerne. Und – […] deswegen hat das für mich was von einer Versuchung. Ja!“

Hier verschränken sich zwei Dynamiken: Zum einen das Abarbeiten gegenüber den Vorurteilen, zuhause nicht oder weniger zu arbeiten – ein Vorurteil, mit dem Frauen stärker konfrontiert sind, weil ihnen unterstellt wird, sich im Homeoffice eher um Familien- und Hausarbeitsaufgaben zu kümmern –, zum anderen die interessierte Selbstgefährdung (Peters 2011), d. h. die Bereitschaft, auch gesundheitsschädigende Mehrarbeit bewusst in Kauf zu nehmen.

### Geschlechts(un)typische Zuschreibungen als Ermöglichungen und Hinderungsgründe für neue Arrangements

In den Interviews finden sich verschiedene Plausibilisierungen und Rechtfertigungen für die jeweilige Arbeitsteilung und die Entscheidung, wer orts- und zeitflexibel arbeitet. Ein Deutungsmuster bezieht sich auf das Stereotyp, Frauen könnten im Gegensatz zu Männern mehr Dinge parallel machen. Eine Angestellte beschreibt dies folgendermaßen:

Ich könnte mir vorstellen, dass es für Frauen ein besseres Angebot ist, weil die mehr parallel machen dann. Also ich glaube, dass das für Männer eher so ein unangenehmeres Thema sein könnte. […] Also ich hätte mir das jetzt auch bei meinem Mann nicht so gut vorstellen können, dass er von zu Hause aus arbeitet und dann nebenher mal eben hier mal eben die Wäsche anwirft oder so was. Also ne, das sind die Kleinigkeiten, die bekommt man hin. Oder mal kurz ein Telefonat privat annimmt. […] Das sind so Kleinigkeiten, da – natürlich macht man das. Oder dem Postboten die Tür aufmacht oder hier und da was macht. In der eigenen Mittagspause macht man ja das Essen fürs Kind. Also ich weiß gar nicht, ob das jedem so angenehm ist.

Demgegenüber beschreibt eine weibliche Führungskraft sich selbst als rastlos und ihren Mann als viel geduldiger mit den Kindern, weshalb sie deutlich mehr arbeitet als er, und er freiberuflich sehr viel im Homeoffice ist – und damit auch die Hauptverantwortung für die Kinder hat:

Ja, ich glaube, dass – ja, mein Wille, dass – also diese Karriere machen zu wollen, hatte ich eigentlich immer. […] ich bin – es muss immer weitergehen. Stillstand kann ich gar nicht leiden. […] Jetzt zum Beispiel, der Kleine ist ja noch ein Baby und ich war – hatte einen Monat Elternzeit genommen. […] Und mein Mann, der kann das auch einfach viel besser, der hat eine riesen Geduld. Ich bin nicht, wie gesagt, so eine – Geduld habe ich nicht, ne?

Eine andere weibliche Führungskraft betont, dass sie das Homeoffice unter keinen Umständen für die Betreuung der Kinder nutzen würde, sondern ausschließlich um konzentriert zu arbeiten. Sie antwortet auf die Frage, ob sie mittags im Homeoffice kochen würde: „Nein. Nein. Nein. […] Und wenn ich Homeoffice mache, dann = dann – also das lege ich eigentlich auch so zu den Tagen, dass sie [die Kinder] dann nicht mittags da sind. […] Also da muss ich schon ein bisschen gucken. […] Ja, wenn ich arbeite, dann arbeite ich.“

## Diskussion

Die Analyse orts- und zeitflexiblen Arbeitens aus Geschlechterperspektiven verweist auf verschiedene Effekte: Zunächst zeigt sich tatsächlich, dass sich der Vereinbarkeitsstress, den viele tagtäglich im Spannungsverhältnis zwischen beruflichen und familiären Anforderungen erfahren, verringert: Möglichkeiten orts- und zeitflexiblen Arbeitens führen zu zeitlichen und emotionalen Entlastungen und Entspannungen bei der Vereinbarkeit von Beruf und Familie. Zudem zeigen sich Ansätze für eine leichte Annäherung beim Zuverdiener*innen-Modell: Betriebliche Angebote für Homeoffice ermöglichen (weiblichen) Teilzeitbeschäftigten, ihre vertraglich vereinbarte Arbeitszeit zu erhöhen – und damit Einkommen, Rente sowie Entwicklungsmöglichkeiten. So befördert orts- und zeitflexibles Arbeiten materielle Sicherheit und verringert den gender gap hinsichtlich der Arbeitszeit.

Dabei verändert sich aber oftmals nicht die Verteilung unbezahlter Haus- und Sorgearbeit, so dass vor allem Frauen mit Hilfe digitaler Technologien und flexibler Arbeitsarrangements insgesamt mehr Arbeit leisten, bezahlt, unbezahlt, und dies auch in Form unsichtbarer Überstunden, ohne dass dies betrieblich und gesellschaftspolitisch sichtbar wird. Zugespitzt befördern digitale flexible Angebote damit die individualisierte Alltagsoptimierung und führen zu einer Verunsichtbarung der dadurch entstehenden Anforderungen und Belastungen. Der Arbeitsumfang insgesamt nimmt möglicherweise zu. Die Frage, die hier zu diskutieren wäre, ist, ob das Ziel orts- und zeitflexiblen Arbeitens tatsächlich sein sollte, Rahmenbedingungen zu schaffen, in denen alle so viel Erwerbsarbeit wie möglich leisten.

Deutlich wird auch, wie wirkmächtig die Rahmenbedingungen für die praktizierte Arbeitsorganisation und -teilung sind. Das Beispiel des Vaters, der im Gegensatz zu seiner Frau flexibel arbeiten kann, und sich daher mehr um die Kinder kümmert, wenn er im Homeoffice ist, zeigt, wie groß der Einfluss betrieblicher Regelungen, Vereinbarungen und Angebote zu orts- und zeitflexiblem Arbeiten auf die Alltagsorganisation in Familien sein kann.

Insgesamt wird deutlich, wie verschiedene Bedingungen für die Frage nach Vereinbarkeit und Arbeitsteilung zusammen wirken: Die Art und Weise, wie der Alltag (mit Kindern) gestaltet wird, hängt stark von den Ansprüchen an den Alltag (Zeit mit Kindern) und an die Erwerbsarbeit (Job gut machen wollen) sowie von Rollenbildern (mein Mann könnte das nicht; mein Mann ist viel geduldiger mit den Kindern, Idealvorstellungen von Muttersein/Vatersein) ab. Genauso deutlich ist aber auch geworden, dass der Entscheidung, wie die Haus- und Sorgearbeit aufgeteilt wird, oftmals ein gewisser Alltagspragmatismus zugrunde liegt, bei dem Wegezeiten und die Möglichkeiten, die der Job anbietet, wichtige Faktoren sind. Angebote für orts- und zeitflexibles Arbeiten können dann unter Umständen tatsächlich zu einer Verringerung der Ungleichheit in der geschlechterdifferenzierenden Arbeitsteilung führen. Neben mittlerweile sehr guten, stabilen und komfortablen technologischen Möglichkeiten sind wichtige förderliche Bedingungen vor allem betriebliche Rahmenbedingungen, Regelungen und Kulturen, verständnisvolle Vorgesetzte sowie auch gleichstellungspolitisch motivierte und motivierende Betriebsrät*innen und Führungskräfte, die orts- und zeitflexibles Arbeiten als Lösung für Vereinbarkeitsprobleme ernsthaft gestalten und umsetzen. Inwiefern dabei wiederum die aktuellen, durch die Corona-Pandemie bedingten Dynamiken im Homeoffice noch eigene, spezifische Effekte haben werden, die Ungleichheiten zu verschärfen oder abzubauen, bleibt letztlich abzuwarten und bedarf weiterer Forschungen.
